# Precise functional connections between the dorsal anterior cingulate cortex and areas recruited for physical inference

**DOI:** 10.1111/ejn.15670

**Published:** 2022-05-09

**Authors:** Ana Navarro‐Cebrián, Jason Fischer

**Affiliations:** ^1^ Department of Psychological and Brain Sciences Johns Hopkins University Baltimore MD USA; ^2^ Department of Psychology University of Maryland College Park MD USA

**Keywords:** anterior cingulate cortex, functional connectivity, intuitive physics, resting‐state fMRI, social reasoning

## Abstract

Recent work has identified brain areas that are engaged when people predict how the physical behaviour of the world will unfold—an ability termed *intuitive physics*. Among the many unanswered questions about the neural mechanisms of intuitive physics is where the key inputs come from: Which brain regions connect up with intuitive physics processes to regulate when and how they are engaged in service of our goals? In the present work, we targeted the dorsal anterior cingulate cortex (dACC) for study based on characteristics that make it well‐positioned to regulate intuitive physics processes. The dACC is richly interconnected with frontoparietal regions and is implicated in mapping contexts to actions, a process that would benefit from physical predictions to indicate which action(s) would produce the desired physical outcomes. We collected resting state functional magnetic resonance imaging (MRI) data in 17 participants and used independent task‐related runs to find the pattern of activity during a physical inference task in each individual participant. We found that the strongest resting state functional connections of the dACC not only aligned well with physical inference‐related activity at the group level, it also mirrored individual differences in the positioning of physics‐related activity across participants. Our results suggest that the dACC might be a key structure for regulating the engagement of intuitive physics processes in the brain.

## Graphical abstract

We studied the dorsal anterior cingulate cortex (dACC) as a candidate for regulating the engagement of intuitive physics mechanisms in service of our goals. We collected resting state fMRI data and independently used a physical inference task to localize the regions active during physical prediction in each participant. We found that the pattern of dACC functional connectivity precisely tracked with individual differences in task‐related activity, with an individual's own functional connectivity map being the best predictor of where their activity would emerge during the physical prediction task.

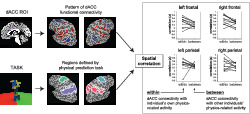

**Abbreviation list**
ACC: anterior cingulate cortex
dACC: dorsal anterior cingulate cortex
fMRI: functional magnetic resonance imaging
FWHM: full width at half maximum
MD: multiple demand
MNI: Montreal Neurological Institute
MVPA: multivariate pattern analysis
ROI: region of interest

## 1 BACKGROUND

**FIGURE 1:**
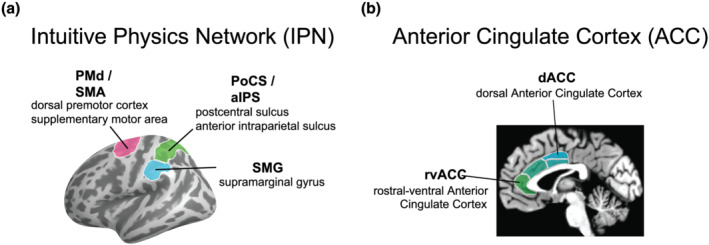
Brain regions engaged by physical prediction tasks (panel a) and the anterior cingulate cortex (ACC; panel b). Both the physical prediction‐related activity and the ACC are shown here on a single hemisphere but are present bilaterally

People can form rapid and accurate intuitions about the physical structure of everyday scenes and the way their physical dynamics will play out in the immediate future. For example, we can use estimates of the weight of a water pitcher, the volume of the water inside and the three‐dimensional shapes of the water pitcher and glass to rapidly and implicitly predict how the water will flow when the pitcher is tipped, adjusting our actions to pour a glass of water without spilling a drop. Our intuitive understanding of how the physical dynamics will play out in such scenarios is termed *intuitive physics* (Fischer, 2020; Kubricht et al., 2017). Recent work investigating the brain regions recruited for intuitive physics uncovered areas in dorsal parietal and frontal cortex that are engaged more during physical prediction than other difficulty‐matched tasks (Figure 1a; Fischer et al., 2016)—specifically, bilateral dorsal premotor cortex (PMd)/supplementary motor area (SMA), bilateral postcentral sulcus (PoCS)/anterior intraparietal sulcus (aIPS), and the left supramarginal gyrus (SMG). Additional work has shown that the same regions are recruited when inferring latent physical properties from observed events (Schwettmann et al., 2019), and damage within these areas can yield impairments in mechanical reasoning alongside the movement difficulties characteristic of motor apraxia (Goldenberg & Hagmann, 1998; Goldenberg & Spatt, 2009). Thus, these regions may be the neural locus of operations supporting intuitive physics.

At the same time, the brain regions recruited for physical prediction show notable overlap with other established networks in the dorsal stream (Fischer et al., 2016). In particular, there is a close apparent alignment with networks associated with tool use and action planning (Gallivan & Culham, 2015), as well as the multiple demand (MD) network which comprises a set of flexible resources that can be deployed across a broad array of tasks (Duncan, 2010; Duncan & Owen, 2000). Unlike some functional modules that exhibit a high degree of domain‐specificity (Kanwisher, 2010), the neural machinery of intuitive physics appears to be embedded within a broader landscape of cognitive processes supporting executive function and action preparation. With the study of the neural mechanisms of intuitive physics still nascent, there is a long way to go to understand the interplay among these co‐localized systems, and this work proceeds in parallel with behavioural studies on the separability of intuitive physics from other facets of cognition (Fischer, 2020; Mitko & Fischer, 2020). One possibility is that physical prediction draws at least in part on flexible multiple demand resources. While the activity of the MD network as a whole scales with difficulty on a wide variety of tasks, there is also heterogeneity within the network with different tasks eliciting reliably different patterns of response (Stiers et al., 2010). Studies of functional connectivity have also revealed subnetworks within the MD system (Camilleri et al., 2018; Stiers et al., 2010). Subregions of the MD network (particularly those associated with action planning) may carry out computations that are particularly well‐suited to the structured ruleset and online dynamics of physical scenarios. Still, it is worth noting that the brain regions recruited for physical inference do not overlap perfectly with the MD network, as shown by split‐half correlation analyses (Fischer et al., 2016). This underscores the need for further work to more thoroughly characterize the neural substrates of intuitive physics, including their functional connections.

An important clue to understanding how intuitive physics fits within the family of functions associated with dorsal frontoparietal cortex will come from investigations of functional connectivity, uncovering the brain areas that reliably co‐activate with those recruited for physical prediction. We approached the present study with an eye towards the kinds of information that would be important for physical prediction in everyday environments. Visuospatial areas no doubt provide critical inputs to intuitive physics mechanisms—information about objects' geometry, surface properties and velocities, to name a few. Beyond these inputs providing a spatial scene description, there should also be mechanisms for regulating the deployment of physical prediction to specific elements of a scene. In everyday scenes, we are constantly surrounded by a multitude of physical interactions happening simultaneously. For example, our dog could be about to knock over a valuable object while we are trying to pour a glass of water. If we cannot analyse all physical interactions in a complex environment at once, we must have a mechanism that dictates the current focus of physical prediction according to our goals, expertise and motivations, as well as the novelty and salience of the physical content in the world. What brain structures might mediate this selection process? Based on two complementary lines of evidence, we see the anterior cingulate cortex (ACC; Figure 1b) as a prime candidate for directing physical prediction processes towards goal‐relevant scene contents. The first line of evidence comes from the putative function of the ACC in linking context‐sensitive goals to appropriate actions, and the second line of evidence comes from the established functional connectivity of the anterior cingulate with areas nearby or overlapping those recruited for physical inference.

First, a theory of ACC function holds that it operates as a switchboard for linking contexts to strategies (Heilbronner & Hayden, 2016) or translating intentions to actions (Paus, 2001). For example, the dorsal anterior cingulate (dACC) would represent contexts and task variables relevant for behaviour and link those with the appropriate strategies for action (Heilbronner & Hayden, 2016). In that sense, the dACC would be more active in situations in which the appropriate behaviours in a specific context are not known or rehearsed and willed control of action is required (Paus, 2001). In line with this notion, based on evidence from task‐ and rest‐based functional connectivity, Camilleri et al. (2018) proposed that the pre‐SMA/medial cingulate cortex node of the multiple demand network might orchestrate the engagement of other multiple demand regions (such as those found in physical prediction tasks) based on task demands. This function is crucial in the context of physical prediction. In a soccer game, for example, the strategy or action of a goalkeeper will involve coordinated movements of the arms, hands and other body parts to impede the opposing team's attempt to score. To act as a switchboard, the ACC would connect various inputs (i.e., a ball approaching) to outputs (i.e., use of different body parts), and these links between input and output would be modulated by internal factors such as motivation and goals (i.e., different goals and motivations of a goalkeeper and a striker).

Second, a number of prior functional connectivity studies have established that the dorsal ACC co‐activates with regions nearby or overlapping the ones recruited for intuitive physics during tasks and at rest. For example, studies with rhesus monkeys have shown that the dorsal ACC projects to premotor cortex (Pandya et al., 1981) and receives input from posterior parietal lobe among other regions (Vogt & Pandya, 1987). Additionally, functional connectivity studies in humans have shown that the activity of the dorsal ACC correlates with sensorimotor areas (Habas, 2010; Margulies et al., 2007; Yu et al., 2011).

Additionally, functional specialization has been described within the ACC, with the rostral‐ventral aspects of the ACC being associated with tasks that involve social cognition and dorsal aspects associated with tasks that require spatial cognition (Bush et al., 2000; Mao et al., 2017; Palomero‐Gallagher et al., 2019; Somerville et al., 2006; Vogt et al., 1992). Patterns of connectivity from the ACC mirror this organization; for example, whereas rostral‐ventral parts of the ACC are connected to areas important for social cognition, the dACC is well‐connected—both anatomically and functionally—to regions of the frontal and parietal cortex that are important for spatial cognition (Devinsky et al., 1995; Habas, 2010; Margulies et al., 2007; Yu et al., 2011). Based on these functional characteristics, the dACC would be ideally suited for routing selected scene contents to physical prediction mechanisms based on the context, goals and motivations.

The above findings notwithstanding, there has yet to be a direct test of the functional coupling between the ACC and brain regions recruited for physical prediction, especially in the fine‐grained way afforded by running functional localizers in individual participants. Here, we tested the functional connectivity of the ACC using an individual differences approach inspired by recent findings showing that an individual's functional connectivity ‘fingerprint’ can be used to predict the patterns of activity that will be observed during tasks (Tavor et al., 2016; Tobyne et al., 2017, 2018). These studies, along with others investigating anatomical connections with diffusion‐weighted imaging (Saygin et al., 2012, 2016), have shown that individual differences in the pattern of task‐related brain activity reflect underlying patterns of connectivity intrinsic to the individual. We reasoned that if we found reliable individual differences in the pattern of activity observed during a physical prediction task, we could leverage those individual differences to test whether the functional connections of the dACC precisely track with the brain areas recruited for physical prediction or whether the dACC simply connects in a non‐specific way with the same general neighbourhood of the multiple demand network. Analysing the spatial correlation between ACC connectivity and task‐related fMRI responses, we found that an individual's own pattern of dACC functional connectivity best predicted their task‐related activity. This finding further advances the dACC as a candidate for orchestrating the deployment of physical prediction processes.

## 2 METHODS

### 2.1 Subjects

Seventeen subjects participated in an functional magnetic resonance imaging (fMRI) experiment that lasted around 90 min total. All subjects gave written informed consent in accordance with the Institutional Review Board at Johns Hopkins University. They were right‐handed, English native speakers, and none of them had a history of neurological or psychiatric illnesses. Ages ranged from 18 to 35 years. Gender information was only recorded for 9 of the 17 participants (6 of these 9 were female). All subjects were compensated for their participation. Subjects started by completing a series of screening questionnaires. After that, the participants went through a maximum of 16 scans: Two of them were resting state scans, and the remaining functional runs contained a variety of tasks, most of which are not reported on here. An intuitive physics task (described below) and the resting state scans are the focus of the present study. From the initial 17 subjects, 1 subject was not included because an anatomical lesion was detected.

### 2.2 MRI acquisition

Structural and functional MRI data of the whole brain were collected on a 3 Tesla Phillips scanner. A *T*_1_‐weighted high‐resolution (1 × 1 × 1 mm) anatomical image (MPRAGE) was collected for each subject. Functional data were collected using a *T*_2_*‐weighted echo planar imaging pulse sequence (TR = 2 s; matrix size = 80 × 80; voxel size = 3 × 3 × 3.5 mm).

### 2.3 Resting state scans

During each of the 5‐min resting state scans, the lights in the scanning room were dimmed, and participants were shown a blank black screen. They were instructed to keep their eyes open during the scan and look at the screen but were otherwise given no instructions on what to think about during the scan. One resting state run was collected near the beginning of the scanning session, and the other was collected at the end. Two participants did not have time to complete any resting state scans, and two additional subjects were excluded of the resting state connectivity analyses due to excessive movement during the resting state scanners. Four of the participants included in the analyses only had time for one resting state scan of 5 min.

### 2.4 Task

**FIGURE 2:**
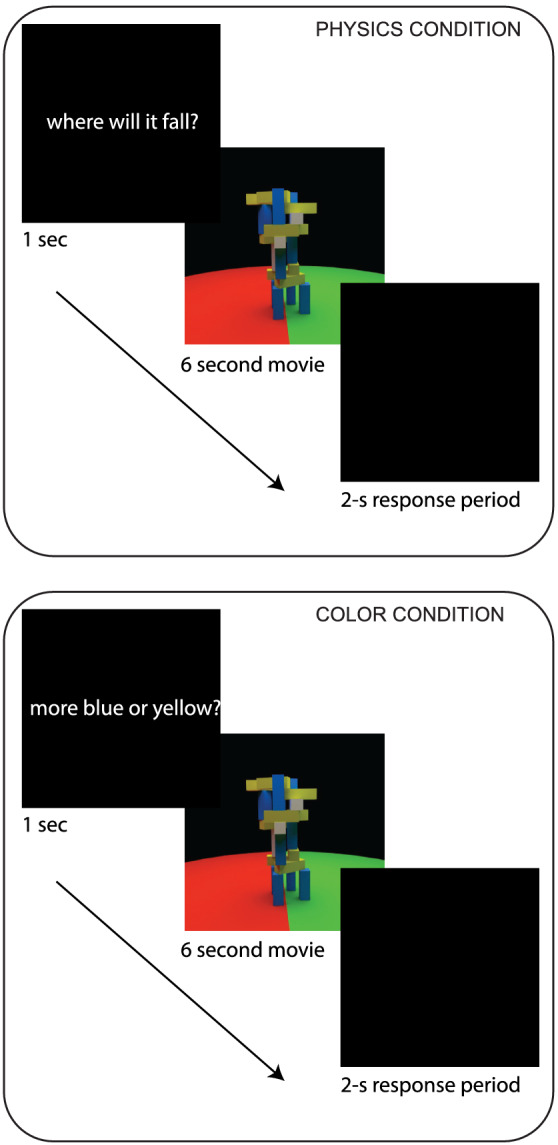
Example of the experimental task: Physical versus colour judgements with identical stimuli. After the text cue indicating the type of judgement to perform (physical or colour), participants viewed a video of the tower rotating in 360° that allowed them to see the arrangement from all sides. Each video was followed by a 2‐s response period

The task (Figure 2) used here to localize regions recruited for physical prediction is identical to the one in Fischer et al. (2016). Participants viewed videos of unstable block towers and were asked to judge either where the blocks would land if the tower tumbled (physical judgement) or whether the tower contained more blue or yellow blocks (colour judgement). The stimuli presented for the two tasks (physical and colour judgments) were visually identical, and the tasks were matched on difficulty. Each participant completed two runs of this task. Stimuli were based on those used by Battaglia et al. (2013) and were created in Blender 2.70 (Blender Foundation; https://www.blender.org).

Each fMRI scanning run consisted of 23 blocks of 18 s each: 10 blocks of the physical judgement task, 10 blocks of the colour judgement task and 3 rest blocks, which consisted of only a blank black screen. Each nonrest block began with a text cue, displayed for 1 s, which read either ‘more blue or yellow?’ (colour task) or ‘where will it fall?’ (physical task). The cue was followed by a 6‐s movie clip of a block tower, with the camera panning to show the tower from all sides. Each movie clip was followed by a black screen during a 2‐s response period. This sequence was repeated twice within a block, with the same task being cued for both movie presentations within a block. Rest blocks occurred in blocks 1, 12 and 23, and the nonrest blocks were arranged in a pseudorandom palindromic order, so that the pairwise ordering between block types was balanced across a run. A scanning run lasted for 414 s (207 volumes with a 2‐s TR).

### 2.5 Resting state image processing and connectivity analysis

Preprocessing of resting state fMRI data was performed using the AFNI software package (http://afni.nimh.nih.gov). Standard preprocessing procedures were used (afni_proc.py) including despiking, slice‐time correction and coregistration. Images were smoothed with a 4‐mm full width at half maximum (FWHM) Gaussian kernel and warped to standardized Talaraich space. TRs with greater than 2 mm of movement were censored and removed. Data were band‐pass filtered using standard cut‐offs (0.009 < *f* < 0.08; i.e., Kayser et al., 2012). Motion parameters and their derivatives (roll, pitch, yaw, dS, dP, dL) as well as white matter and ventricular timeseries were included as regressors of no interest.

Our analyses focused primarily on dACC regions of interest (ROIs) in the left and right hemisphere, obtained from the human Brainnetome atlas, a connectivity‐based parcellation that provides a functional subdivision of the human brain (Fan et al., 2016). This posterior part of the ACC is also referred to as the midcingulate cortex (Margulies & Uddin, 2019; Procyk et al., 2016; Shackman et al., 2011; Shenhav et al., 2016; Vogt, 2016; Vogt et al., 2003). We also conducted connectivity analyses in 6 additional ROIs (3 in the left hemisphere and 3 in the right) that covered the remaining extent of the ACC (rostral‐ventral and caudal‐dorsal), for the sake of comparison with the dACC. We used FreeSurfer (https://surfer.nmr.mgh.harvard.edu/) to map the Brainnetome atlas to each individual subject. The time series of all the ROIs' common variance was regressed out from the preprocessed data in order to ensure that the connectivity for each parcel reflected its unique variance. While regressing out mean signal can introduce spurious relationships in resting state data in certain circumstances (Murphy et al., 2009), our interest here was specifically in the spatial distribution of dACC functional connectivity and its overlap with intuitive physics regions. Regressing out common variance among the subregions of the ACC in this case allowed us to examine the precise targets of dACC functional connections separately from the general connectivity of the ACC (Margulies et al., 2007). We performed a correlation analysis using AFNI to calculate the functional connectivity between each of these brain regions and all other brain voxels. Correlation values were *z*‐transformed, and we performed a one sample *t* test against zero on the *z* scores. Corrections for multiple comparisons were carried out by applying a cluster‐size correction derived from the AFNI programs 3dFWHMz (using a mixed model) and 3dClustsim on data thresholded at a value of *p* < 0.001.

Next, we calculated the overlap between the active regions from the physical prediction task and the voxels whose activity correlated positively with the ACC ROIs. For the intuitive physics areas, we used a group of masks from Fischer et al. (2016) that were shown to be selectively engaged when people predicted the unfolding of physical events. These ROIs are cortical parcels, defined to include the extent of cortex in standardized space over which the majority of individual subjects' peak activations would be expected to fall (Julian et al., 2012). They cover larger extents of cortex than any one individual's intuitive physics regions would, but they are ideally suited as ROIs for the purposes of this present study. We first used these parcels to evaluate whether the strongest functional connections of the dACC fall within the expected neighbourhood of the regions engaged by the physical prediction task (described below in Section 2.6). We then used these parcels to constrain our multivariate pattern analyses, allowing for individual subjects' physics‐related responses, which varied in their cortical location, to fall within the regions of cortex used for the correlation analyses (described below in Section 2.8).

### 2.6 Group‐level analysis (resting state connectivity)

To compute the general overlap of the peak dACC functional connectivity with the regions active during physical inference at the group level, we first applied an increasingly stringent threshold on the ACC correlation results and created a group of 20 datasets with the highest percentile values, starting at the 99th percentile and ending at the 100th percentile at steps of 0.05. For each of these 20 datasets, we calculated the percentage of voxels that overlapped with significant voxels from the physical prediction task within a mask from the Talairach Daemon atlas that covered frontal and parietal lobes.

### 2.7 Intuitive physics task preprocessing and analysis

Data preprocessing was conducted using afni_proc.py and consisted of despiking, slice‐time correction, coregistration, smoothing and warped to standardized Talairach space. TRs with greater than 3 mm of movement were excluded from analysis. Data were band‐pass filtered (0.009 < *f* < 0.08). Motion parameters and their derivatives (roll, pitch, yaw, dS, dP, dL) were included as regressors of no interest. We used the cue phases of the task for the two different conditions (towers' physics and towers' colour) as separate regressors. Regressors were convolved with a gamma haemodynamic response function peaking at 4.7 s. One participant with an anatomical lesion and another participant with excessive movement during the task were not included in the analysis.

### 2.8 Correlation‐based subject specificity analysis

To analyse whether the connectivity of the dACC could predict individual differences in the pattern of activity during physical prediction, we calculated the spatial correlation (correlation across voxels) for each individual between the resting state connectivity of the dACC and that individual's physics–colour contrast. Correlation analyses were conducted within functionally defined physics' ROIs, obtained from the significant activity during the physical prediction task, as described below. For each individual, we also calculated the average of the correlations between their resting state connectivity of the dACC and the physics–colour contrast for all the other participants. We then conducted an analysis of variance (ANOVA) on the correlation values to test for a main effect of within‐ versus between‐subject correlations, which would indicate that dACC connectivity tracks individual differences in physics‐related activity.

An independent test was performed to analyse whether we could expect to have sufficient power to find reliable individual differences in the pattern of dACC functional connectivity within our sample of participants in the main study. For this purpose, we used a non‐published sample of 10 subjects with 5 min of resting‐state fMRI data collected at the University of California, Berkeley. Data were preprocessed using the same steps described above for the resting‐state analyses of the present study. The preprocessed resting‐state data were divided into two halves, and the connectivity of the left and right dACC and the regions engaged in physical inferences was calculated for each half. After converting the connectivity results to *z* values, we calculated the correlation across voxels (spatial correlation) of the first half with the second half. We then converted these within‐subject correlations (first half correlated with second half) to *z* values and compared them with the between‐subject spatial correlations (first half of one individual correlated with the average of the first half of all other individuals). A repeated‐measures ANOVA revealed significant differences between within‐ and between‐subject correlations; *F*_1,9_ = 51.9, *p* = 0.000051, with significantly higher within‐subject spatial correlations and overall higher spatial correlations in the left hemisphere (*F*_1,9_ = 6.82, *p* = 0.028). The interaction between these two factors was not significant (*p* = 0.077). These results revealed statistically reliable individual differences in the pattern of dACC functional connectivity with the dorsal frontoparietal cortex. This result gave us confidence that our main study had sufficient power to characterize individual differences in dACC functional connectivity and find a link with intuitive physics task‐related activity, if one exists.

### 2.9 Physics versus colour contrast

After excluding one subject due to excessive movement during the physics task, 15 participant‐specific contrast (physics–colour) images for the right and left dACC were entered into one sample *t* tests. Corrections for multiple comparisons were carried out by applying a cluster‐size correction derived from the AFNI programs 3dFWHMz (using a mixed model) and 3dClustsim on data thresholded at a value of *p* < 0.001 (uncorrected).

## 3 RESULTS

**FIGURE 3:**
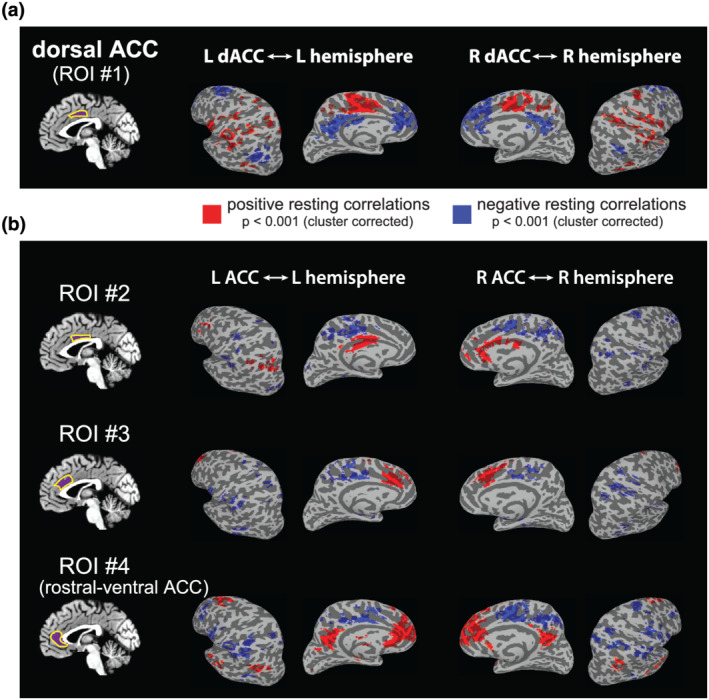
Group‐level resting state functional connectivity maps (12 subjects). (a) Functional connectivity of the dorsal anterior cingulate cortex (ACC), our primary target for investigation. Significant positive resting correlations are shown in red (*p* < 0.001, cluster‐corrected), and significant negative resting correlations are shown in blue (*p* < 0.001, cluster‐corrected). (b) Functional connectivity for three additional subdivisions of the ACC, shown here for comparison with the dorsal anterior cingulate cortex (dACC). Colour coding thresholds are the same as in (a)

Prior studies have reported that the dACC shares functional connectivity with portions of the frontal and parietal cortex (Margulies et al., 2007; Yu et al., 2011), with networks important for attention allocation, implementation of task‐sets and motor planning, among other (Amodio & Frith, 2006; Beckmann et al., 2009; Hutchison et al., 2012; Margulies & Uddin, 2019; Neubert et al., 2015; Torta & Cauda, 2011). We first sought to test, at the group level, the degree to which these connections actually overlap with previously reported locations active during intuitive physics tasks (Fischer et al., 2016). We conducted a functional connectivity analysis using four parcels of the ACC in each hemisphere from the Brainnetome atlas as ROIs. Our interest was in whether the general pattern of dACC functional connectivity in fronto‐parietal cortex matched what had been previously reported as the intuitive physics network (Fischer et al., 2016), and the remaining parcels of the ACC served as comparison cases to confirm that their functional connections are concentrated elsewhere. Figure 3 shows the patterns of functional connectivity for the dACC (Figure 3a) and the remaining subdivisions of the ACC (Figure 3b). In line with prior findings (Margulies et al., 2007), the dACC showed robust connectivity with regions of frontal and parietal cortex in the vicinity of the regions engaged in physical inferences, setting the stage for a quantitative assessment of how closely these functional connections align with task‐defined intuitive physics regions.

**FIGURE 4:**
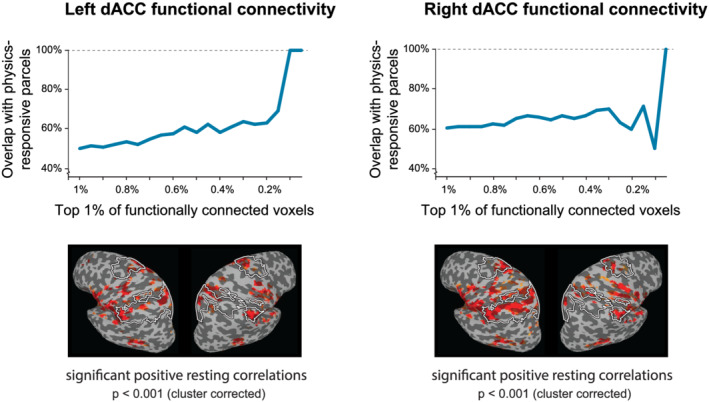
Overlap between regions identified with the intuitive physics localizer and the voxels whose activity correlates positively with the dorsal anterior cingulate cortex (dACC). The proportion of voxels falling within the intuitive physics parcels is plotted as a function of the threshold applied to the functional connectivity maps, with increasingly stringent thresholds plotted as one moves rightward along the abscissa. The degree of overlap increased with successively stringent thresholds, ultimately reaching 100% overlap for the functional connectivity of both the left‐ and right‐hemisphere dACC. The intuitive physics parcels are outlined in white on the functional connectivity maps below, which show all significant (*p* < 0.001, cluster‐corrected) positive correlations

To quantify the overlap between the peak locations of dACC connectivity (Table 1) and the areas engaged in physical inferences, we first performed a group‐level analysis, comparing the functional connectivity with the published locations of these regions active during intuitive physics tasks (Fischer et al., 2016). Figure 4 shows the proportion of the most strongly dACC‐connected voxels that fall within the expected location of the regions engaged in physical inferences. We found that when examining the top 1% of voxels showing the strongest resting correlations with the dACC, 50–60% of the voxels fell within the physics regions. As we applied successively more stringent thresholds on the functional connectivity map, the degree of overlap with the physics regions rose more or less monotonically, up to 100% overlap at the most stringent thresholds. This pattern of results indicates that at least at the group level, the voxels with the very strongest functional connections with the dACC fall within the regions engaged in physical inferences.

**TABLE 1:** MNI coordinates of clusters most significantly correlated with right and left dorsal ACC in parietal areas

	MNI coordinates			
	*X*	*Y*	*Z*	*p* value, uncorrected
Right ACC connectivity	−46	−42	52	<0.0001
Left ACC connectivity	12	−57	64	<0.0001

*Note*: The coordinates represent the maximum value within the cluster.

Abbreviations: ACC, anterior cingulate cortex; MNI, Montreal Neurological Institute.

**FIGURE 5:**
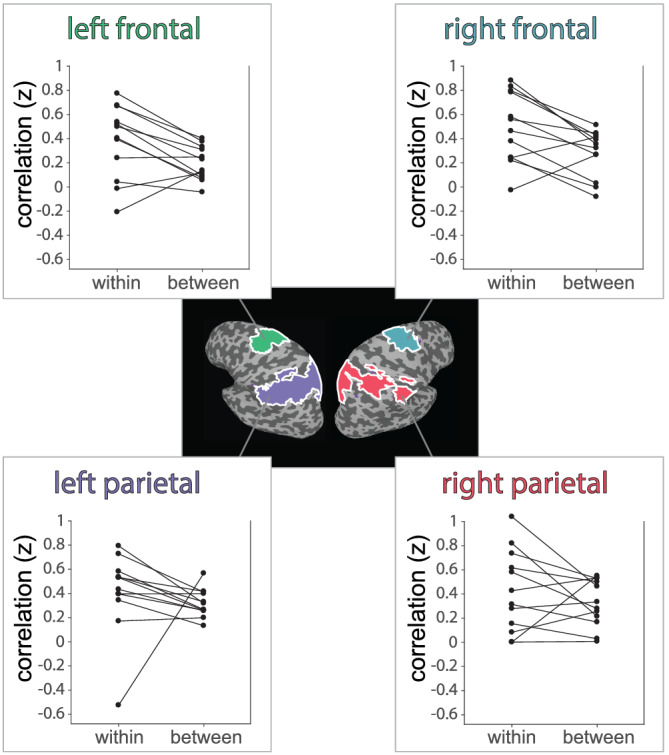
Within and between‐subject spatial correlations (correlations across voxels) between the resting state connectivity of the dorsal anterior cingulate cortex (ACC) and the physics–colour contrast. The connected points on each plot show a participants ‘within’ correlation (the pattern of their dorsal anterior cingulate cortex (dACC) connectivity compared with their own pattern of task‐related activity) and their corresponding ‘between’ correlation (the same participants dACC connectivity compared with the task‐related activity of other participants). We entered the full set of these correlation values into an analysis of variance (ANOVA) analysis to test for a main effect of within‐ versus between‐subject correlations

If the peak functional connections of the dACC specifically target the most active areas from the physical prediction task as suggested by the group‐level analysis, then we should observe that the best‐connected voxels align with physical prediction task‐related activity on an individual subject basis. To test this hypothesis, we conducted a correlation‐based multivariate pattern analysis (MVPA), computing the spatial correlation for each individual between the resting state connectivity of the dACC and the physics–colour contrast (within‐subject correlation). We compared the within‐subject correlation to a between‐subject correlation, comparing each subject's pattern of dACC functional connectivity with each of the remaining individual subject's physics‐related activity. The between‐subject correlation for a particular participant was taken as the average of the *z*‐scored correlations for that participant's connectivity map with each of the remaining individual task‐related maps. This analysis tested whether an individual's pattern of dACC connectivity better predicted their own physics‐related responses than the physics‐related activity of others in the group—if dACC functional connections simply land in the neighbourhood of the regions important for physical intuition but do not directly target them, then we would not expect the spatial pattern of dACC connectivity to reliably correlate better with an individual's own task‐related activity than with other subjects' task‐related activity. Figure 5 shows the within‐ versus between‐subject correlations for four ROIs (frontal and parietal regions in both the left and right hemisphere) engaged in physical intuition. Within‐subject correlations were reliably higher than between‐subject correlations, as revealed by an ANOVA (a significant main effect of within‐ vs. between‐subject; *F*_4,8_ = 14.82, *p* = 0.0078, with no significant main effect of region [*p* = 0.50] and no significant interaction [*p* = 0.63]). This correlation‐based pattern analysis established that the pattern of the dACC's resting‐state connectivity reliably predicted an individual subject's pattern of task‐related activity when making physical predictions. Additionally, prior work has shown that motion in the scanner can produce spurious functional connectivity despite regression of motion parameters (Power et al., 2012). For this reason, we tested the possibility that the spatial correlation effects between dACC connectivity and physics activity were correlated with the amount of motion in the scanner. The correlation across‐subjects was found to be not significant (Pearson's *r* = 0.19).

Lastly, we considered the possibility that dACC functional connections actually target the brain regions associated with other aspects of cognition such as action planning or spatial attention, which engage brain networks that at least partially overlap with the regions engaged in physical inferences (Fischer et al., 2016). While we did not localize action planning or spatial attention‐related brain regions in our participants, the physical prediction task that we used was controlled for attentional demands and did not explicitly require any action planning (besides button presses, which were matched across conditions). Thus, activity in our dACC, ROI during the physical prediction task would point to an engagement in intuitive physics over and above these other cognitive functions. To test this possibility, we evaluated the physics  colour contrast within the dACC ROI in the left and right hemispheres. For the contrast, we used the 3dttest++ AFNI function. A statistical threshold of *t* = 3.3 was employed, corresponding to a corrected *p* < 0.005. Five voxels survived that threshold and clustering in the left dACC ROI and 34 voxels were significant in the right dACC. The significant response in the dACC during physical prediction relative to a well‐matched control condition points to its engagement with the intuitive physics system rather than adjacent and overlapping attention and action systems. Note that this does not imply, nor do we mean to suggest, that the dACC is part of a network engaged in physical intuition or directly involved in intuitive physics computations. Rather, we take these data to align with existing ideas about ACC function—when a person's current goals require physical inferences about some scene elements, the dACC engages the areas engaged in intuitive physics on those scene elements, providing an interface between intentions and the processes needed to advance them. The dACC responds during physical prediction, engaging the regions necessary for physical intuition, and other subregions of the ACC engage other cognitive systems during the non‐physics control condition.

Lastly, in agreement with previous data (Margulies et al., 2007), the connectivity analyses show that the dACC has negative correlations with medial prefrontal areas and posterior cingulate cortex (PCC), which are regions involved in social reasoning. These regions have been reported to have a mutually inhibitory relationship with the intuitive physics system in the brain (Jack et al., 2013). On the other hand, the activity of the most rostral‐ventral part of the ACC analysed (ROI #4; Figure 3) correlates with medial prefrontal areas and PCC among others and shows negative correlations with the dorsal ACC, operculum as well as parietal and frontal areas previously shown to activate during intuitive physics tasks (Fischer et al., 2016). We interpret these negative correlations with caution, because mean signal regression can introduce apparent anti‐correlations between networks (Murphy et al., 2009). The raw dACC signal may not be anti‐correlated with the resting state activity social brain regions, but the key point here is that the dACC resting signal is *less correlated* with social regions than with the rest of the brain, and likewise, rostral‐ventral ACC signal is less correlated with brain regions engaged in physical than with much of the rest of the brain. These results suggest that the rostral‐ventral ACC may play a complementary role to the dACC, engaging social processes in a goal‐directed fashion (see Section 4).

## 4 DISCUSSION

We spend much of our daily life making predictions about the interaction of objects in our environment. For example, we are constantly calculating the speed of moving things, the distance to obstacles or the strength we need to apply to open, close or lift an object. All of these computations happen automatically and without apparent mental effort. Recent studies have identified a set of frontal and parietal brain regions that are engaged when people predict the unfolding of physical events. Here, we show that the functional connections of the dACC precisely target these regions recruited for physical inferences, tracking individual differences in the location of the voxels that are most engaged during physical prediction. This link between the dACC and the intuitive physics system in the brain fills in a missing piece of the puzzle in understanding the function of these brain areas. The ACC, for example, is believed to be an interface between goals and actions, engaging behaviours that best suit the current context (Heilbronner & Hayden, 2016). But what specific actions would help achieve the current goals? If I want to move a stack of items from one table to another, what specific actions should I take? Intuitive physics provides the answer, serving as a means of evaluating the likely physical outcomes of possible actions. The intuitive physics system might say that the stack is too unstable to move all at once, so the appropriate action under physical constraints is to divide the stack in two and move each separately. Without an assessment of stability, the appropriate action might seem to be lifting the entire stack at once. In this light, our findings here augment the current understanding of both the intuitive physics system and the anterior cingulate, establishing the dACC as a strong candidate for regulating the engagement of mental physics.

As discussed in Fischer et al. (2016), the frontal and parietal brain areas engaged when people make physical inferences overlap with the multiple‐demand network (MDN; Duncan, 2010), especially with those parts relevant for action planning. The nature of the interaction among these overlapping systems remains to be characterized, and the answer could take the form of a completely unified account (e.g., physical predictions being accomplished by a subset of the flexible resources within the MDN), a multiplexing of independent systems within common cortical real estate or an intermediate case that affords some degree of interaction between distinct systems by virtue of their co‐localization. The latter account has some appeal from an ecological perspective. Nearly all actions that we take are informed by expectations about their physical outcomes, so much so that it scarcely makes sense to have an action planning system without accompanying physical prediction mechanisms, whatever their format. Physical prediction might be the ‘flip side of the coin’ of action planning, representing the physical consequences of a family of possible actions from which an action plan samples the most desirable. At the same time, physical prediction itself is both a highly structured and highly variable challenge whereby a limited set of physical principles can be applied to understand a vast array of scenarios. Flexible mental programmes in the MDN might help accommodate these heterogeneous scenarios while capitalizing on the structured nature of the problem. It is notable that the ACC has also been implicated in the MDN (Camilleri et al., 2018; Duncan, 2010), and its activity during executive function tasks could reflect the recruitment other regions of the MDN when these are needed (Camirelli et al., 2017). Our final result showed a good match between resting state and task networks, and as previous research has noted (Cole et al., 2014; Smith et al., 2009; Tavor et al., 2016), this may indicate that brain areas within functional networks are continuously interacting even at rest.

The possible role of the dACC in intuitive physics also accords with theories that implicate the ACC in mapping contexts to options and strategies (Heilbronner & Hayden, 2016; Holroyd & Yeung, 2012) or mapping intentions to actions (Paus, 2001). In that sense, dorsal parts of the ACC may activate specifically when, based on the context, motivation and goals, higher attention to certain physical properties of objects is needed as well as the proper actions in response to those physical stimuli. For example, when we see that a glass of water is about to fall from a table, the activity of the dACC would be required to recruit the appropriate sensory and motor areas to avoid that happening. This idea falls in line with studies that have suggested that the extensive anatomical connections between the cingulate gyrus and premotor and parietal areas could provide a basis for ‘limbic’ influences on motor and spatial attention mechanisms respectively (Pandya et al., 1981; Vogt & Pandya, 1987).

We have used a connectivity‐based parcellation of the ACC for our analysis in which voxels within each parcel share similar brain connectivity. It is possible that a different parcellation would give us different connectivity patterns. One possibility worth testing is that finer‐scaled structure within the dACC could map to different task properties and stimuli. For example, different kinds of physical inferences (inferring weight vs. predicting dynamics) might map to different parts of the dACC and corresponding frontoparietal cortex. Additionally, a limitation in resting‐state connectivity studies can be the effect of previous tasks (Grigg & Grady, 2010; Hasson et al., 2009; Tambini et al., 2010; Wang et al., 2012). As explained in Section 2, one of the resting state scans was collected at the beginning of the scanning session, and another was collected at the end, after a series of intuitive physics tasks. Although we cannot rule out the possibility that the connectivity of the dACC was affected by the previous intuitive physics tasks, it is important to note that this effect cannot account for the individual differences observed in our spatial correlation analyses.

In addition to the dACC, we analysed the connectivity of other parcels of the ACC. In agreement with previous literature (Margulies et al., 2007), the functional connectivity of the most rostral‐ventral part of the ACC appears to overlap with brain regions that are important for social cognition. This finding agrees with studies that show that different parts of the ACC activate with different types of actions (Bush et al., 2000; Mohanty et al., 2007; Stevens et al., 2011). Specifically, the rostral‐ventral ACC and dorsal ACC seem to be involved in emotional and cognitive functions, respectively (Bush et al., 2000). Previous studies (Baron‐Cohen et al., 1986, 2001) have differentiated two aspects of causal cognition: intuitive physics, or the ability to understand and predict the physical dynamics of our everyday environment, and intuitive psychology, or the ability to understand and predict others' thoughts and behaviours. Jack et al. (2013) suggested that there is reciprocal inhibition between the brain networks that underlie intuitive physics and intuitive psychology or social cognition. In their findings, when people performed a mechanical reasoning task, areas associated with social reasoning had reduce activity. On the other hand, when people engaged in a social reasoning task, brain areas associated with mechanical reasoning had reduce activity. In agreement with previous fMRI data (Margulies et al., 2007), our analyses show opposite patterns of connectivity for the rostral‐ventral ACC and dorsal ACC. Because the ACC has been suggested to be a task switcher (Cocuzza et al., 2020) or a selector of appropriate actions (Heilbronner & Hayden, 2016), depending on the goals and context, the activation of the dorsal ACC may prioritize physics actions over social ones, and the activity of the rostral‐ventral ACC may prioritize social actions over physics ones.

An important note is that while intuitive physics and social cognition engage distinct networks in the brain, distinctness does not necessarily imply independence or competition between these systems. Indeed, there are many cases where physical inference rests on social perception (Zhang et al., 2019) and social inferences rest on physical understanding (Liu et al., 2017), so an interaction between these systems mediated by the ACC would not strictly be a competitive one. Rather, it would reflect an optimizing of resources for the present goals, which may sometimes manifest as a trade‐off between systems as in Jack et al. (2013) and sometimes as a cooperation between systems as the situation demands.

Finally, although we have focused in this article on the study of the most dorsal part of the ACC due its connectivity to the areas engaged in physical intuition, the functional parcellation that we used includes several other parts of the ACC that connect to networks other than the physics and social ones mentioned above. Previous studies offer some hints about the brain areas associated to these other parts of the ACC and their roles in behaviour (Margulies et al., 2007). One possibility is that the anatomy of the ACC follows a hierarchy similar to the one studied more generally in the frontal lobe (Badre, 2008; Badre et al., 2010; Badre & D'Esposito, 2007, 2009; Badre & Nee, 2018), going from concrete to abstract types of action representations as we move from dorsal to rostral‐ventral areas. Future experiments may study this hierarchy specifically in the ACC and see how this fits the cognitive/emotional dichotomy of the ACC (Bush et al., 2000).

In sum, our findings point to the dACC as a prime target for future study in the context of physical prediction. The study of the intuitive physics system in the brain is still in its infancy, and these results provide a promising link to other well‐studied systems for connecting intentions to actions.

## CONFLICT OF INTEREST

Authors report no conflict of interest.

## AUTHOR CONTRIBUTIONS

AN‐C and JF performed the conceptualization, analysis of data and drafting of manuscript.

### PEER REVIEW

The peer review history for this article is available at https://publons.com/publon/10.1111/ejn.15670.

## Data Availability

fMRI data will be made freely available via the lab's website at the time of publication. IPN localizer stimuli are available on the lab website, and all presentation and analysis code are available upon email request to the authors, with no restrictions.
